# Supplementary material for: P-TRAP: a Panicle Trait Phenotyping tool
Source: BMC Plant Biol. 2013 Aug 29;13:122. doi: 10.1186/1471-2229-13-122 (PMC3848748; doi:10.1186/1471-2229-13-122)
Supplement: Additional file 7 — P-TRAP source code. The Netbeans project that contains the source code of the application. [file 1471-2229-13-122-S7.zip › javadoc.zip!/javadoc/org-basmati-workspace/org/basmati/workspace/EditandViewGrainFileActionDataObject.html]

EditandViewGrainFileActionDataObject (WorkSpace)


JavaScript is disabled on your browser.


- Package
- Class
- Tree
- Deprecated
- Index
- Help

*org.basmati.workspace 1.0*

- Prev Class
- Next Class

- Frames
- No Frames

- All Classes

- Summary:
- Nested |
- Field |
- Constr |
- Method

- Detail:
- Field |
- Constr |
- Method


org.basmati.workspace

## Class EditandViewGrainFileActionDataObject

- java.lang.Object
- - org.openide.loaders.DataObject
  - - org.openide.loaders.MultiDataObject
    - - org.basmati.workspace.EditandViewGrainFileActionDataObject

- All Implemented Interfaces:
  :   java.io.Serializable, org.openide.nodes.Node.Cookie, org.openide.util.HelpCtx.Provider, org.openide.util.Lookup.Provider

  ---

    

  ```
  public class EditandViewGrainFileActionDataObject
  extends org.openide.loaders.MultiDataObject
  ```

  The data object implementation to represent the grain result file.

  Author:
  :   faroqal-tam

  See Also:
  :   `MultiDataObject`,
      Serialized Form

- - ### Nested Class Summary

    - ### Nested classes/interfaces inherited from class org.openide.loaders.MultiDataObject

      `org.openide.loaders.MultiDataObject.Entry`
    - ### Nested classes/interfaces inherited from class org.openide.loaders.DataObject

      `org.openide.loaders.DataObject.Container, org.openide.loaders.DataObject.Factory, org.openide.loaders.DataObject.Registration, org.openide.loaders.DataObject.Registrations, org.openide.loaders.DataObject.Registry`
  - ### Field Summary

    - ### Fields inherited from class org.openide.loaders.DataObject

      `PROP_COOKIE, PROP_FILES, PROP_HELP, PROP_MODIFIED, PROP_NAME, PROP_PRIMARY_FILE, PROP_TEMPLATE, PROP_VALID`
  - ### Constructor Summary

    Constructors

    Constructor and Description

    `EditandViewGrainFileActionDataObject(org.openide.filesystems.FileObject pf,
    org.openide.loaders.MultiFileLoader loader)`
  - ### Method Summary

    Methods

    | Modifier and Type | Method and Description |
    | --- | --- |
    | `protected int` | `associateLookup()` |
    | `static org.netbeans.core.spi.multiview.text.MultiViewEditorElement` | `createEditor(org.openide.util.Lookup lkp)` |

    - ### Methods inherited from class org.openide.loaders.MultiDataObject

      `addSecondaryEntry, createNodeDelegate, files, findSecondaryEntry, getCookie, getCookieSet, getHelpCtx, getLookup, getMultiFileLoader, getPrimaryEntry, handleCopy, handleCopyRename, handleCreateFromTemplate, handleDelete, handleMove, handleRename, isCopyAllowed, isDeleteAllowed, isMoveAllowed, isRenameAllowed, registerEditor, registerEntry, removeSecondaryEntry, secondaryEntries, setCookieSet, takePrimaryFileLock`
    - ### Methods inherited from class org.openide.loaders.DataObject

      `addPropertyChangeListener, addVetoableChangeListener, copy, createFromTemplate, createFromTemplate, createFromTemplate, createShadow, delete, dispose, find, firePropertyChange, fireVetoableChange, getCookie, getFolder, getLoader, getName, getNodeDelegate, getPrimaryFile, getRegistry, handleCreateShadow, isModified, isShadowAllowed, isTemplate, isValid, markFiles, move, removePropertyChangeListener, removeVetoableChangeListener, rename, setModified, setTemplate, setValid, toString, writeReplace`
    - ### Methods inherited from class java.lang.Object

      `clone, equals, finalize, getClass, hashCode, notify, notifyAll, wait, wait, wait`

- - ### Constructor Detail


    - #### EditandViewGrainFileActionDataObject

      ```
      public EditandViewGrainFileActionDataObject(org.openide.filesystems.FileObject pf,
                                          org.openide.loaders.MultiFileLoader loader)
                                           throws org.openide.loaders.DataObjectExistsException,
                                                  java.io.IOException
      ```

      Throws:
      :   `org.openide.loaders.DataObjectExistsException`
      :   `java.io.IOException`
  - ### Method Detail


    - #### associateLookup

      ```
      protected int associateLookup()
      ```

      **Overrides:**
      :   `associateLookup` in class `org.openide.loaders.MultiDataObject`


    - #### createEditor

      ```
      public static org.netbeans.core.spi.multiview.text.MultiViewEditorElement createEditor(org.openide.util.Lookup lkp)
      ```


- Package
- Class
- Tree
- Deprecated
- Index
- Help

*org.basmati.workspace 1.0*

- Prev Class
- Next Class

- Frames
- No Frames

- All Classes

- Summary:
- Nested |
- Field |
- Constr |
- Method

- Detail:
- Field |
- Constr |
- Method
